# Chemical compositions, contaminants, and residues of organic and conventional goat milk in Bogor District, Indonesia

**DOI:** 10.14202/vetworld.2019.1218-1224

**Published:** 2019-08-09

**Authors:** Veronica Wanniatie, Mirnawati B. Sudarwanto, Trioso Purnawarman, Anuraga Jayanegara

**Affiliations:** 1 Department of Animal Infectious Diseases and Veterinary Public Health, Graduate School of Veterinary Public Health, IPB University, Bogor, Indonesia; 2 Department of Animal Husbandry, Faculty of Agriculture, University of Lampung, Indonesia; 3 Department of Animal Diseases and Veterinary Public Health, Faculty of Veterinary Medicine, IPB University, Bogor, Indonesia; 4 Department of Nutrition and Feed Technology, Faculty of Animal Science, IPB University, Bogor, Indonesia

**Keywords:** antibiotic, chemical composition, goat milk, heavy metal, pesticide

## Abstract

**Aim:**

This study aimed to compare chemical composition and contaminants (pesticide residues, antibiotic residues, and heavy metal residues) between organic and conventional goat milk in Bogor District, West Java Province, Indonesia.

**Materials and Methods:**

Milk sampling was carried out from March to August 2018 at six goat farms. The chemical quality of milk was checked using the Lactoscan Ultrasonic Milk Analyzer device. Fatty acids were analyzed using gas chromatography (GC). Pesticide residues in goat’s milk were analyzed using a GC-electron capture detector (GC-ECD). Antibiotic residues were analyzed using bioassay screening test method. The lead (Pb) and arsenic (As) residues were analyzed using the Atomic Absorption Spectrophotometer (AAS).

**Results:**

The content of fat, protein, and lactose showed that there was no difference in the composition of goat’s milk between organic and conventional farms. Caprylic acid (C8:0) and capric acid (C10:0) of organic goat milk are higher than conventional goat milk. Stearic acid (C18:0) and linoleic acid (C18:2) of conventional goat milk are higher than organic goat milk. The total fatty acid of organic goat milk is higher than conventional goat milk. Organochlorine pesticide residues were not detected in organic goat milk and conventional goat milk. Tetracycline antibiotic residues were found in one sample (5.56%) of organic goat milk, and macrolides residues were found in two samples (11.11%) of conventional goat milk. Pb residue in organic goat milk is 50 ppb while conventional goat milk is 80 ppb. Residue As in organic goat milk is 70 ppb while conventional goat milk is 110 ppb.

**Conclusion:**

There was no chemical composition (fat, protein, and lactose) difference between organic and conventional goat milk. Saturated fatty acid (SFA) in organic goat milk is higher than conventional goat milk. Pesticide residues are not found in both organic and conventional goat milk. Tetracycline antibiotics were found in organic goat milk and macrolide antibiotic groups found in conventional goat milk. Pb and As residues were found in both organic goat milk and conventional goat milk.

## Introduction

Milk contains many essential nutrients, so it is recommended to be consumed regularly by children. Consumers are looking for food that can improve their health. One kind of milk that can be used as food with good nutritional value is organic milk. Organic milk has a higher selling value than milk derived from the conventional farming system because it implements high requirements regarding quality in the production and management process [1].

Nowadays, the demand for organic milk is increasing [2,3] assuming that consuming milk from organic farms will provide different benefits compared to consuming milk from conventional farms [3]. Milk prices derived from organic farms are higher than milk originating from conventional farms because organic milk is produced environmentally friendly from livestock that does not use antibiotics, hormones, synthetic chemicals, and without genetic modification so that it has potential benefits for human health [4]. The nutritional content of organic milk differs from conventional milk [5], while other reports claim that there is no difference [6,7].

Organic goat milk consumed cannot be guaranteed to be free from various contaminants. Contaminants can come from feed or the environment around the cage. Contaminants which can contaminate milk are pesticide residues, antibiotics, and heavy metals. The presence of antimicrobial residues from a public health standpoint raises a variety of problems, including, the main potential for consumers [8]. Food chemical contamination is an extensive topic of many exogenous chemicals that may or may not be harmful to consumers. In general, contaminants can be categorized as agrochemicals (especially residues of veterinary drugs and pesticides), environmental contaminants (especially heavy metals, persistent organic pollutants, and natural poisons), and processing of contaminants (from cooking, processing, or packaging) [9].

Hazardous chemicals that might contaminate milk can come from the environment, namely, pesticides, antibiotics, and heavy metals. The presence of pesticide residues [10], antibiotics [8], and heavy metals [11] when viewed from a public health perspective, among them, is the main potential for consumer health problems. Pesticides are one of the agrochemical materials used to control pests in plants and animals. Pesticides are a concern of the community because they include dangerous chemical compounds. Excessive use and not following the rules of use can lead (Pb) to agent resistance, residues in food products, and public health disorders such as poisoning, immunosuppressive, and cancer [10].

Antibiotics are used on farms not only for clinical purposes but also as a growth promotor in increasing livestock production. Improper usage of antibiotics can Pb to the resistance of pathogenic bacteria and contributes to the global health crisis. The presence of antimicrobial residues in milk can cause drug hypersensitivity reactions to consumers, such as dermal reactions, asthma, or anaphylaxis [12].

Heavy metals are found widely in the environment and have two primary sources, namely, human activity and geological background. Heavy metals are metallic, and metalloid chemical elements have high atomic weights and specific gravity, which can be toxic to living things. Types of heavy metals in food are arsenic (As), cadmium (Cd), mercury (Hg), tin (Sn), and Pb. Heavy metal content in milk can come from plants or water consumed by livestock [13]; this will cause a buildup of metals in the body and will migrate to humans who consume their products. Indonesian National Standard (SNI) number 3141.1:2011 [14] requires a maximum limit of heavy metal content in milk, namely, Pb of maximum 20 ppb, maximum Hg of 30 ppb, and maximum As of 100 ppb. Pb and As are the most dangerous heavy metals that are carcinogenic and hematopoietic disorders and can cause kidney and gastrointestinal disorders [10,11].

In Indonesia, most consumers prefer to drink raw goat milk because of the belief in better taste and nutritional value and are useful as health-enhancing drugs or even disease healing agents [15]. Besides being a source of nutrition, milk can also contain dangerous chemical contamination. Research on nutrition and chemical contamination in organic goat milk has never been done in Indonesia. This research aimed to compare nutritional values and chemical contamination (pesticide residues, antibiotic residues, and heavy metal residues) between organic and conventional goat milk in Bogor District, West Java Province, Indonesia.

## Materials and Methods

### Ethical approval

No live animals were used in the present study. No ethical approval was needed for the current study.

### Study area

The study was conducted in Bogor District, Indonesia, consist of three location organic farming, i.e., Ciampea (-6.5866710 S’latitude, 106.6843140 W’longitude), Caringin (-6.696547 S’latitude, 10.835356 W’longitude), and Cijeruk (-6.695693 S’latitude, 106.770186 W’longititude) and another three for conventional farming, namely, Ciampea (-6.5644079 S’latitude, 106.6951570 W’longititude), Caringin (-6.730147 S’latitude, 106.834260 W’longititude), and Cijeruk (-6.6976560 S’latitude, 106.7968700 W’longititude).

### Sampling

Milk sampling was carried out from March to August 2018. Samples were taken from six goat farms (three organic farms and three conventional farms), as many as 500 mL from each farm in Bogor District, Indonesia. The samples were put into a sterile bottle and carried using ice cubes at a temperature of 4-10°C. Each milk sample was divided into five, one for chemical analysis using the Lactoscan Ultrasonic Milk Analyzer (Milkotronic, Bulgaria) device, one for determination of fatty acid profiles by gas chromatography (GC), one for organochlorine pesticide residues with GC-electron capture detector (GC-ECD), one for antibiotic residues with bioassay screening test method, and one for metal residues weight with an Atomic Absorption Spectrophotometer (GTA 120 Graphite Tube Atomizer; 200 series AA, Agilent Technologies).

### Procedures

### Milk compositions

Examination of the chemical quality of organic milk was done using parameters such as lactose content, protein content, fat content, moisture content, and nonfat dry matter. A chemical examination was carried out using the Lactoscan Ultrasonic Milk Analyzer (Milkotronic, Bulgaria) device. Fatty acids were analyzed using GC. Before the hydrolysis and esterification process was carried out, fat extraction of milk samples was done using goldfish extraction. After esterification into fatty acid methyl ester (FAME), the sample was analyzed using GC. A standard solution of 1 mg was added to 100 mL of milk sample. Then, 0.5 N NaOH-Methanol solution of 1.5 mL was put into the milk sample and then vortexed. The N_2_ gas was then exhaled to remove O_2_ gas, which can oxidize FAME so that the measurement results will be negative. The mixture solution was put into a water bath with a temperature of 80-100°C for 5 min so that the saponification reaction occurs. The mixture was added with 2 mL of BF_3_-Methanol for the esterification reaction, then blown again with N_2_ gas and put back into the 80-100°C water bath for 5 min. The FAME mixture obtained was separated by solvent extraction (liquid-liquid extraction) by adding hexane as much as 1.5 mL and vortexed. If there was no separation, then 3 mL of saturated NaCl was added and vortexed. Then, the hexane phase was taken carefully and added anhydrous Na_2_SO_4_ to bind water. The liquid formed was then taken using a pipette and injected into the GC (7890A GC System, Agilent Technologies, USA) instrument.

#### Organochlorine pesticide residue test

Analysis of pesticide residues in goat milk using GC (7890A GC System, Agilent Technologies, USA) followed the QuECheRS (Quick Easy Cheap Effective Rugged Safe) method [16]. The 15 g goat milk sample was put into a 50 mL centrifuge tube (Agilent Technologies, USA), and 15 mL of acetonitrile containing 1% acetic acid was added, and QuECheRS salt (Magnesium sulfate, Sodium Acetate, QuECheRS AOAC, Agilent Technologies, USA) and ceramic (Agilent Technologies, USA) were added and then shaken for 1 min to homogenize all parts of the material. The sample was extracted by shaking the homogenizer tube for 5 min. Extraction was continued by centrifuging for 13 min at 14°C at 4000 rpm. The extract was taken as much as 6 mL and put into a dispersive SPE tube (Agilent Technologies, USA) of 15 mL with ceramic then shaken for 2 min then left for 1 min. Then, the extract was centrifuged for 10 min at 4000 rpm. As many as 1-mL supernatants were taken into the evaporation flask to evaporate to dryness. The residue was added with 1 mL of acetone and put into a vial of 1.5 mL. As many as 10 µl of the solution was ready to be injected into the GC-ECD for the detection of pesticide residues.

#### Antibiotic residue test

Antibiotic residues were analyzed using triple bio screening test method, which refers to SNI No. 2782.1998 [17]. The stages of the test consist of preparation, testing, and reading the results. Preparation included preparation of agar media, media culture, buffer solution, and standard solution. Bioassay testing was aimed at four classes of antibiotics, namely, tetracycline, macrolides, aminoglycosides, and penicillin. Media culture was used *Bacillus stearothermophilus* ATCC 7953, yeast extract, peptone, bacto agar, and dextrose for penicillin. Media culture was used *Bacillus cereus* ATCC 11778, yeast extract, beef extract, peptone, and bacto agar for tetracycline. Media culture was used *Bacillus subtilis* ATCC 6633, beef extract, peptone, and bacto agar for aminoglycosides. Media culture was used *Kocuria rhizophila* (*Micrococcus luteus*) ATCC 9341, yeast extract, beef extract, peptone, bacto agar, and glucose for macrolide. A total of 10 mL of sample was put in a test tube.

Meanwhile, media culture was prepared by pouring 8 mL on each Petri dish. Sterile disc paper was then placed on the surface of the media culture. Each Petri dish contained five pieces of paper discs, which consist of three pieces of disc each with 75 µL samples to be analyzed, one paper dripped 75 μL of standard antibiotic solution 0.01 IU/mL as a positive control and one more paper buffer solution phosphate as a negative control. The disc paper was placed on the surface of the media culture. Petri dishes are closed and incubated at different temperatures depending on the antibiotic group. The media culture for the tetracycline group was incubated at 30±1°C and penicillin group at 55±1°C, while the macrolides and aminoglycosides at 36±1°C, for 16-18 h, respectively. Results of the test were done by observing and measuring the diameter of the zone of resistance formed around the paper disc using the calipers. The sample was positive for antibiotics if the inhibition zone was formed ≥2 mm from the edge of the paper disc. The sample was negative if the inhibitory zone formed was 0-2 mm, because the inhibition zone formed <2 mm was considered due to the presence of natural inhibitors. The diameter of the resistance zone in the positive control was 20±1 mm, while the negative control did not form an inhibitory zone.

#### Heavy metal residue test

Determination of Pb and As levels of heavy metals in milk was analyzed through the extraction process using microwave high-temperature dewatering after the process of ignition, precipitation, and dissolution using HNO_3_ and H_2_O_2_ as oxidizers followed by measurements using an Atomic Absorption Spectrophotometer/AAS (GTA 120 Graphite Tube Atomizer; 200 series AA, Agilent Technologies, USA) with a wavelength of 283.3 nm. The work procedure carried out to test Pb and As heavy metal residues were goat milk samples weighed 0.3-0.5 g and put in a sample tube (vessel), then added 65% HNO_3_ as much as 8 mL and 30% H_2_O_2_ with use a 10.0 mL pipette. The test was continued by conducting destruction according to the Microwave Digestion System (Ethos One, Milestone, Italy) program for ±2 h. The results of destruction were transferred into a 50 mL measuring flask and added distilled water thinner to the limit. The dilution results were inserted into the AAS tool to measure the absorbance. Testing of Pb and As heavy metals were done by calibrating the curve by entering the Pb (Lead standard solution, Merck KGaA, Germany) and As (Arsenic standard solution, Merck KGaA, Germany) standards into the AAS system. The Pb and As standards were entered using concentrations of 0.1 µg/g, 0.2 µg/g, 0.3 µg/g, 0.4 µg/g, 0.5 µg/g, and 0.6 µg/g. After obtaining a standard curve, then the filtrate sample was entered into the AAS system. The reading of the test results was done by integrating the results of sample absorbance with the standard heavy metal calibration curve [17].

### Statistical analysis

Using SPSS Statistics version 21.0 (IBM, USA), statistical analysis was performed to explore any differences between organic and conventional milk. Mann–Whitney U-test was used for a quantitative variable, while the exact descriptive test was used for quantitative. Normality of dependent variable and residual was assessed using either Kolmogorov–Smirnov test or Shapiro–Wilk test without showing deviation from normality. The result was considered significantly different statistically (p<0.05) [18].

## Results

The test results on the chemical and physical composition of organic and conventional goat milk are presented in Table-1. The results of statistical tests on the testing of fat, protein, and lactose content showed no differences in the composition of goat milk between organic and conventional farms. Organic and conventional goat milk fatty acids are shown in Table-2. Caprylic acid (C8:0) and capric acid (C10:0) of organic goat milk are higher than conventional goat milk. Stearic acid (C18:0) and linoleic acid (C18:2) of conventional goat milk are higher than organic goat milk. The total fatty acid of organic goat milk is higher than conventional goat milk.

**Table 1:** Chemical and physical composition of organic and conventional goat milk.

Milk composition	Organic (n=18)	Conventional (n=18)
Fat (%)	6.22±1.38	6.15±1.03
Protein (%)	3.51±0.33	3.53+0.20
Lactose (%)	3.45±0.37	3.37±0.19
Density (%)	1,03±0.002	1.03±0.002
pH	6.64±0.088^a^	6.59±0.079^b^
Freezing point	−0.424±0.044	−0.424±0.031

a,b Different superscript within the same row indicate a significant difference (p<0.05).

**Table 2:** Composition of fatty acid in organic and conventional goat milk (mean±SD).

Type of fatty acid (FA)	Organic (% from total FA) (n=18)	Conventional (% from total FA) (n=18)
Caprylic acid (C8:0)	1.53±1.04^a^	1.04±0.15^b^
Capric acid (C10:0)	3.69±1.91^a^	2.66±1.44^b^
Lauric Acid (C12:0)	5.51±4.87	4.07±3.54
Myristic acid (C14:0)	7.49±4.46	6.01±1.91
Palmitic acid (C16:0)	24.68±4.72	24.89±4.99
Stearic acid (C18:0)	19.76±6.33^a^	24.87±7.25^b^
Palmitoleic acid (C16:1)	0.39±0.46	0.49±1.00
Oleic acid (C18:1)	29.90±7.61	31.44±5.08
Linoleic acid (C18:2)	2.62±1.50^a^	3.70±1.79^b^
Linolenic acid (C18:3)	0.48±0.58	0.33±0.45
SFA	62.67±10.05^a^	38.66±9.42^b^
MUFA	30.29±7.75	31.93±5.19
PUFA	3.09±1.69^a^	4.03±2.04^b^
UFA	33.38±8.29	35.96±6.41
PUFA/SFA	0.05±0.03^a^	0.11±0.07^b^
UFA/SFA	0.55±1.19^a^	1.04±0.61^b^

a,b Different superscript within the same row indicate a significant difference (p<0.05). SFA=C8:0+C10:0+C12:0+C14:0+C16:0+C18:0; MUFA=C16:1+C18:1; PUFA=C18:2+C18:3; UFA=MUFA+PUFA. SFA=Saturated fatty acid, MUFA=Monounsaturated fatty acids, PUFA=Polyunsaturated fatty acids, UFA=Unsaturated fatty acid

Organochlorine pesticide residues were not detected in organic goat milk and conventional goat milk (Table-3). The results of analysis based on filter test on antibiotic residues showed that there was one sample (5.56%) of organic goat milk detected containing tetracycline antibiotics and two samples (11.11%) of conventional goat milk containing macrolide antibiotics (Table-4). Pb residue in organic goat milk was 50 ppb, while conventional goat milk was 80 ppb (Table-4). Residue As in organic goat milk is 70 ppb while conventional goat milk is 110 ppb.

**Table 3:** Organochlorine pesticide residues in organic goat milk and conventional goat milk.

Organochlorine pesticide residue (ppb)	Organic (n=18)	Conventional (n=18)
Linden	nd	nd
Heptachlor	nd	nd
Aldrin	nd	nd
Dieldrin	nd	nd
Endrin	nd	nd
Endosulfan	nd	nd
Dichlorodiphenyltrichloroethane (DDT)	nd	nd

Limit of detection=0.3 ppb, nd=not detected

**Table 4:** Antibiotic and heavy metal residues in organic and conventional goat milk.

Residue	Organic (n=18)	Conventional (n=18)
Antibiotic (%)		
Penicillin	0	0
Tetracycline	5.56	0
Aminoglycosides	0	0
Macrolide	0	11.11
Heavymetals (ppb)		
Lead	50±0.13	80±0.13
Arsenic	70±0.13	110±0.19

## Discussion

### Chemical composition

Statistical results on the testing of fat, protein, and lactose content showed no differences in the composition of goat milk between organic and conventional farms. The result is in agreement with the results of Malissiova *et al*. [3] who found that there were no differences in the composition of fat, protein, and lactose in goat milk from organic and conventional farms in Greece. Feeding management or the type of feed provided provides an opportunity for differences in fat, protein, and lactose content. The type of grass/forage given by organic goat farms is almost the same with conventional goat farms. The only difference is that in conventional farms goat given additional concentrations of tofu pulp, tempeh pulp, and date pulp. Additional concentrates only increase the quantity of goat milk, while the fat content remains lower than organic goat milk, in contrast to research by Tsiplakou *et al*. [19] and Tudisco *et al*. [20].

Saturated fatty acids (SFA), namely, caprylic acid (C8:0) and capric acid (C10:0) organic goat milk statistically show differences with conventional goat milk. Organic goat milk has higher caprylic acid and capric acid than conventional goat milk. This result is different from research by Tsiplakou *et al*. [19]. Caprylic acid and capric acid in milk are of exogenous origin, for example, feed and endogenous [21], which are highly dependent on the availability of the acyl CoA synthetase enzyme in liver tissue and mammary tissue. Caprylic acid and capric acid are short chains of SFA, which are relatively higher in organic goat milk due to differences in feed consumption patterns [19]. Organic goats consume grass and legumes, while conventional goats in addition to forages also consume concentrates.

Stearic acid (C18:0) of organic goat milk is lower than conventional goat milk. Stearic acid originates from exogenous which can be synthesized in tissues and organs of livestock including microbes in the rumen. Stearic acid concentration is higher in conventional goat milk because the feed given is in the form of concentrates, namely, tofu pulp and tempeh pulp. The feed used as a source of long-chain SFA is soybean meal, coconut cake, vegetable oil, olive oil, and coconut oil [21].

Linoleic acid (C18:2) organic goat milk is lower than conventional goat milk. Trans fatty acids in goat milk are the result of microbial activity in the rumen. As one of polyunsaturated fatty acids (PUFA) metabolites, linoleic acid (C18:2) transforms into vaccenic acid (C18:1) and lastly into stearic acid (C18:0) through bio hydrogenation[22]. The value of monounsaturated fatty acid (MUFA) and PUFA of conventional goat milk is higher than that of organic goat milk. These results differ from the results of the study [19,20], which showed that the amount of MUFA and PUFA of organic goat milk was higher than conventional goat milk.

### Organochlorine pesticide residues

The primary sources of organochlorine contamination in milk come from grass and feed, drinking water, soil (partially digested during grazing), and air [23]. These compounds in the animal’s body are easily distributed from the digestive tract and accumulate mainly in the liver, adipose tissue, and milk. Organochlorine if consumed in low doses can endanger health resulting in hormonal disorders, reduced intelligence, fertility disorders, and cancer [24]. Indonesia limits organochlorine residue contamination in milk, namely, lindane (10 ppb), heptachlor (6 ppb), aldrin/dieldrin (6 ppb), endosulfan (4 ppb), and dichlorodiphenyltrichloroethane (20 ppb).

### Antibiotic residue

The presence of antibiotic residues in organic and conventional goat milk is due to the use of antibiotics as control, prevention, and treatment of infections [12]. The use of antibiotics in the mother of goats to treat mastitis and other diseases is a common practice in dairy goat farms. The presence of antibiotic residues is usually due to the use of antibiotics from veterinarian prescriptions and insufficient knowledge about appropriate doses, route of administration, or withdrawal time [25]. The presence of antibiotic residues can also cause obstacles in the processing of other dairy products and will eventually cause antibiotic resistance to pathogenic bacteria so that it will become a global health crisis [26,27].

Tetracycline antibiotics detected in organic goat milk are used as a treatment for mastitis. Tetracycline is used in veterinary medicine as a broad-spectrum antibiotic for the treatment of aerobic and Gram-negative anaerobic bacteria, including *Actinomyces*, *Mycoplasma*, *Rickettsia*, and *Spirochete*. Tetracyclines, including chlortetracycline, are routinely used to prevent and treat mastitis in dairy cows [28]. Macrolides are usually used in the treatment of mastitis in goats [8]. The most commonly used macrolides are erythromycin, spiramycin, and tylosin. Tylosin is used globally as a broad spectrum of antibiotics in veterinary medicine against a variety of Gram-positive and Gram-negative anaerobic and aerobic bacteria [28].

In Indonesia, penicillin, tetracycline, macrolides, and aminoglycosides are the most common antibiotics used in the treatment of mastitis among dairy goat farms. Antibiotic residue testing was carried out using screening tests based on the SNI, namely, penicillin, tetracycline, macrolides, and aminoglycosides as a standard protocol [29]. The next research opportunity is to detect the presence of other antibiotic residues such as lincomycin, clindamycin, and pirlimycin that are widely used in dairy goat farms in throughout the world but are still rarely used in Indonesia.

Bioassay is a screening test to identify antibiotic residues that are widely used all globally and often used in Indonesia because they are easy to use, fast, and relatively inexpensive. This test has a high sensitivity indicated by the detection limit of the minimum concentration of antibiotic residue. The detection limit of beta-lactams, tetracycline, macrolides, and aminoglycosides was 0.00125 ppm, 0.03 ppm, 0.1 ppm, and 0.1 ppm, respectively. According to the SNI, a bioassay is a standard procedure to detect antibiotic residues in the dairy product. The bioassay has the potential to determine a wide range of antibiotics within a single test. The presence of antibiotic residues in the media will inhibit the growth of bacteria. The method is applied routinely in the screening of antibiotics within the milk samples [30]. It will be better if the authors tested the positive screening samples with further confirmation method, such as liquid chromatography-tandem mass spectrometry, but such equipment is not available in the authors’ laboratory.

Globally, the use of antibiotics in organic farms is not allowed. If the antibiotics are used to treat the disease, the organic status of the livestock will be lost and must wait 90 days during the organic maintenance period again. Conventional farms can use antibiotics in the process of healing the disease, but the milk to be sold or consumed must be based on withdrawal time.

### Heavy metal residue

Pb residue in organic goat milk was 50 ppb, while conventional goat milk was 80 ppb (Table-4). These values are above the maximum limit set in the SNI No. 3141.1 2011, which is 20 ppb. The residual Pb of goat’s milk is higher than in Pakistan 30-50 ppb [31] and goat’s milk in Iran (7.37 ppb) [32]. The high amount of Pb residue contamination in organic and conventional goat milk is caused by pollution from the environment. The increase in the number of Pb can be due to the use of phosphate fertilizers for plants, the environment around farms and highways [31].

Heavy metals have been reported to be toxic (cytogenetic) to living organisms [33], which are not decomposed [34] and are in the environment for a long time [30]. Impacts on human health due to exposure to heavy metals are kidney failure, osteoporosis, lung and blood cancer, bone damage, gastrointestinal, hormonal disorders, and metabolic disorders including anemia and excretory loss of enzymes and proteins [35,36]. Livestock is a food source for humans; if feed and its maintenance practices cause contamination with heavy metals, it will be harmful to humans [37,38]. Heavy metals in milk usually come from milk containers, processing, and contaminated water that is used for agriculture, animal feed, and the surrounding environment [39]. Heavy metals commonly found in foods are Hg, As, Cd, and Pb [40].

As residue in organic goat milk is 0.07 mg/kg while conventional goat milk is 110 ppb. Organic goat milk has As residues which are lower than SNI No. 3141.1 2011 (100 ppb), while conventional goat milk has higher As residues. The residue of As in organic and conventional milk is higher than that reported by Rey-crespo *et al*. [36], namely, 1.048 ppb and 0.921 ppb, respectively, in Spain. These results are also higher than the As levels in goat milk in Italy (5 ppb) [40] but lower than in Pakistan (403 ppb) [41]. As is the most toxic metal found in the food chain and is related to cancer cases in humans [28]. The residual content of As in livestock is shown to be an indicator of As content in the soil [42].

## Conclusion

The results indicated that there were no differences in fat, protein, and lactose levels between organic and conventional goat milk. The fatty acid profile produced that caprylic acid, capric acid, and the amount of SFA organic goat milk were significantly different from conventional goat milk. Pesticide residues are not found in both organic and conventional goat milk. Tetracycline antibiotics were found in organic goat milk and macrolide antibiotic groups found in conventional goat milk. Pb residue in organic goat milk and conventional goat’s milk and As residue in conventional goat’s milk were higher than SNI No. 3141.1. 2011.

## Authors’ Contributions

MBS supervised the research. VW carried out the analyses. VW wrote the manuscript with the help of MBS, TP, and AJ. All authors read and approved the final manuscript.
